# The Diagnostic Impact of Contrast-Enhanced Computed Tomography (CECT) in Evaluating Lymph Node Involvement in Colorectal Cancer: A Comprehensive Review

**DOI:** 10.7759/cureus.61832

**Published:** 2024-06-06

**Authors:** Akash Inamdar, Raju K Shinde

**Affiliations:** 1 General Surgery, Jawaharlal Nehru Medical College, Datta Meghe Institute of Higher Education and Research, Wardha, IND

**Keywords:** treatment planning, staging, diagnostic accuracy, lymph node involvement, contrast-enhanced computed tomography (cect), colorectal cancer

## Abstract

Colorectal cancer (CRC) remains a significant global health burden, necessitating accurate staging and treatment planning for optimal patient outcomes. Lymph node involvement is a critical determinant of prognosis in CRC, emphasizing the importance of reliable imaging techniques for its evaluation. Contrast-enhanced computed tomography (CECT) has emerged as a cornerstone in CRC imaging, offering high-resolution anatomical detail and vascular assessment. This comprehensive review synthesizes the existing literature to evaluate the diagnostic impact of CECT in assessing lymph node involvement in CRC. Key findings highlight CECT's high sensitivity and specificity in detecting lymph node metastases, facilitating accurate staging and treatment selection. However, challenges such as limited resolution for small lymph nodes and potential false-positives call for a cautious interpretation. Recommendations for clinical practice suggest the integration of CECT into multidisciplinary treatment algorithms, optimizing imaging protocols and enhancing collaboration between radiologists and clinicians. Future research directions include refining imaging protocols, comparative effectiveness studies with emerging modalities, and prospective validation of CECT's prognostic value. Overall, this review stresses the pivotal role of CECT in CRC management and identifies avenues for further advancements in imaging-guided oncology care.

## Introduction and background

Colorectal cancer (CRC) is a significant global health concern, as it is the third most commonly diagnosed cancer and the second leading cause of cancer-related deaths worldwide. It arises from the malignant transformation of the colon or rectal epithelial cells. The aetiology of CRC is multifactorial, involving genetic, environmental, and lifestyle factors. While the incidence of CRC varies geographically, its prevalence is notably high in developed countries [1]. Lymph node involvement plays a crucial role in determining the prognosis and guiding treatment decisions in patients with CRC. Lymph node metastasis is among the most significant predictors of disease recurrence and overall survival. An accurate assessment of lymph node status is essential for appropriate staging and stratification of patients into risk categories, influencing the selection of adjuvant therapies and surgical management strategies [2].

Contrast-enhanced computed tomography (CECT) is a widely utilized imaging modality in diagnosing and staging CRC. CECT provides high-resolution anatomical images and allows for visualizing vascular structures and tissue perfusion dynamics. The intravenous contrast administration enhances the differentiation between the tumour and normal tissues, facilitating the detection and characterization of lesions. In evaluating CRC, CECT is particularly valuable for assessing locoregional extent, identifying distant metastases, and evaluating lymph node involvement [3]. This review aims to comprehensively examine the diagnostic impact of CECT in evaluating lymph node involvement in CRC. By synthesizing the existing literature, we aim to elucidate the accuracy, reliability, and clinical utility of CECT in this context. Additionally, we will explore the comparative effectiveness of CECT with other imaging modalities, discuss their clinical implications, and highlight future research and clinical practice directions.

## Review

Colorectal cancer: pathophysiology and staging

A Brief Overview of the Pathology of Colorectal Cancer

Colorectal cancer, a slowly developing but preventable malignancy, originates from abnormal cellular changes in the colon or rectal polyps. Despite its preventable nature, it persists as the third most common cancer globally and the second most diagnosed cancer in developed nations. Early detection through screening is paramount, given that up to 65% of the patients fail to complete the recommended screenings. Understanding the obstacles to screening compliance is crucial to curbing colorectal cancer occurrences [4]. The pathophysiology of colorectal cancer entails a multifaceted interplay of genetic mutations, environmental factors, and cellular mechanisms. Most colon cancers stem from adenomas, following the adenoma-to-carcinoma sequence, substantiated by epidemiological, clinical, pathological, and molecular genetic evidence [5]. Defective DNA mismatch repair characterizes a subset of colorectal cancers and is associated with mutations in genes linked to the disease [6]. Colorectal cancer poses a significant global health threat, leading to a considerable number of deaths annually [7].

TNM Staging System

The TNM (tumour, node, metastasis) staging system is the most widely accepted and utilized method for staging solid tumour cancers globally. It categorizes the anatomical extent of such cancers based on three key parameters: the size and extent of the primary tumour (T), the involvement of regional lymph nodes (N), and the presence or absence of distant metastases (M). The T component, representing the tumour, spans from T0 (signifying no detectable primary tumour) to T4 (indicative of extensive local invasion) [8-10]. Meanwhile, the N component, which signifies lymph node involvement, ranges from N0 (denoting no regional lymph node involvement) to N3 (suggestive of extensive lymph node involvement) [8-10]. Lastly, the M component addresses metastasis, with M0 denoting no distant metastasis and M1 indicating its presence [8-10]. By amalgamating these components, an overall stage is assigned, ranging from Stage 0 (carcinoma in situ) to Stage IV (distant metastasis). Higher stage numbers correlate with more advanced cancer. In Stage 0, abnormal cells are localized and have not spread to nearby tissues. In Stages I, II, and III, the cancer is present, with higher numbers indicating larger tumour size and greater spread into nearby tissues. Stage IV signifies cancer that has disseminated to distant parts of the body. Accurate staging is pivotal for tailoring patient management strategies, devising treatment plans, and facilitating meaningful clinical research. By providing a standardized language, the TNM system enables healthcare professionals to communicate effectively regarding a patient's cancer and collaborate on determining the most suitable treatment approach [8-10].

Importance of Lymph Node Involvement in Staging

The significance of lymph node involvement in cancer staging cannot be overstated, as it plays a pivotal role in determining the extent of the disease and guiding treatment decisions. The lymph node status furnishes vital prognostic information and profoundly influences treatment strategies. In cancers such as breast cancer, for instance, the presence or absence of cancer cells in the lymph nodes significantly impacts both prognosis and treatment planning. A node-negative status indicates that cancer has not spread to nearby lymph nodes. In contrast, a node-positive status suggests lymph node involvement, potentially indicating a higher risk of metastasis to distant organs [11,12]. Furthermore, research emphasizes the importance of examining an adequate number of lymph nodes for accurate staging. In gastric cancer, for instance, survival prognostications based on the number of involved nodes are notably more reliable when at least 15 are scrutinized [13]. Similarly, in colorectal cancer, the number of affected lymph nodes assumes critical importance for precise staging, with studies pinpointing a minimum threshold necessary for accurate staging and enhanced survival outcomes [14]. This emphasizes the importance of meticulous lymph node evaluation in ensuring accurate cancer staging and informing optimal treatment strategies.

Contrast-enhanced computed tomography (CECT): principles and techniques

Introduction to Computed Tomography (CT) Imaging

Computed tomography (CT) is a diagnostic imaging modality employing X-ray radiation and computer processing to produce intricate, cross-sectional body images [15-17]. During a CT scan, the CT scanner revolves an X-ray source and detectors around the patient, gathering numerous X-ray projections from diverse angles. A computer subsequently processes these projections to reconstruct the examined region's axial images or "slices" [15-17]. Offering superior clarity and granularity in contrast to conventional X-ray images, CT imaging facilitates the visualization of internal organs, skeletal structures, soft tissues, and vasculature [17]. The patient is gradually conveyed through the circular aperture of the CT scanner on a motorized table while the X-ray source and detectors revolve around them [17]. As the X-ray beam traverses the patient's body, detectors opposite the source record the transmitted X-rays [17]. The data obtained during each rotation is transmitted to a computer, synthesizing individual snapshots into cross-sectional images [17]. These images can be examined individually or compiled to construct a three-dimensional representation of the scanned area [16,17]. CT scans find widespread application in diagnostic, treatment planning, interventional, and screening contexts across various anatomical regions [17]. In essence, CT imaging is a potent diagnostic instrument harnessing advanced X-ray technology and computer algorithms to furnish high-fidelity, detailed depictions of the body's internal structures, facilitating precise diagnosis and treatment strategizing for various medical conditions.

CECT: Mechanism and Agents

CECT operates by employing radiocontrast agents, predominantly iodine-based, to augment the discernibility of structures that would otherwise pose challenges in differentiation from their surrounding milieu [18]. These contrast agents heighten the attenuation of X-rays in the tissues where they accumulate, resulting in augmented signal intensity on the CT images [19,20]. The contrast enhancement mechanism is contingent upon the particular agent utilized and the tissue under examination [19]. For instance, anionic iodinated contrast agents can be employed to evaluate articular cartilage's glycosaminoglycan (GAG) content and biomechanical attributes [19]. Here, the contrast agent aggregates in regions with diminished GAG content, facilitating the detection of early osteoarthritis changes [19]. Predominantly, iodine-based contrast agents are the go-to choice for CT imaging [18]. While these agents are typically administered intravenously, oral and rectal administration may be employed in select cases [21]. The selection of the contrast agent hinges on factors such as the patient's renal function, susceptibility to allergic reactions, and the specific diagnostic query being addressed [21]. For instance, anionic, tri-iodinated contrast agents appraise articular cartilage. In contrast, macrophage-activating nanoparticulate contrast agents find utility in augmenting liver imaging, and lead oxide nanoparticles are deployed for both in vitro and in vivo imaging [22]. The dosage of contrast agent administered is typically calibrated based on the patient's body weight [23]. Standardized protocols are instituted to optimize the timing of imaging and the quality of contrast enhancement in various organs and tissues. CECT harnesses iodine-based contrast agents to amplify the clarity of structures and furnish functional insights into tissues. The contrast enhancement mechanism is contingent upon the specific agent employed and the tissue under scrutiny, with standardized protocols ensuring the optimal timing and fidelity of the images [23].

Technical Parameters for Optimal Lymph Node Evaluation

Optimal lymph node evaluation is critical in cancer diagnosis and staging, ensuring the accurate evaluation of disease extent and assessment of prognosis. Technical parameters for achieving optimal lymph node evaluation entail examining a sufficient number of lymph nodes (ELNs). Studies have delved into determining the ideal number of ELNs across various cancer types, including colon, distal, oesophageal, and rectal cancers [24]. In the context of node-negative colon cancer, research suggests that attaining a minimum of 15 ELNs is associated with enhanced overall survival rates [25]. This recommendation is derived from comprehensive data analyses from the Surveillance, Epidemiology, and End Results (SEER) database and single-centre cohorts, with adjustments for confounding variables. For distal oesophageal cancer, investigations have identified an optimal cutoff value for lymph node dissection number, with a threshold of ≥ 24 ELNs correlating with improved overall survival rates [26]. Furthermore, optimal cutoff values for lymph node metastasis number (1) and rate (0.13) have been delineated in these studies. In the case of rectal cancer patients undergoing neoadjuvant therapy, achieving a lymph node yield of ≥ 12 is deemed necessary for accurate nodal status determination. However, this may not always be feasible [27]. However, research indicates that a lymph node yield of ≥ 10 can still offer enhanced predictive accuracy for survival outcomes, particularly in node-negative patients. The technical parameters for optimal lymph node evaluation involve ensuring the examination of a sufficient number of lymph nodes tailored to the specific cancer type. Recommendations vary across cancer types, with 15 ELNs suggested for node-negative colon cancer, 24 for distal oesophageal cancer, and 10 or 12 for rectal cancer, particularly in the context of neoadjuvant therapy. These recommendations are grounded in robust analyses of large datasets and meticulous control for confounding variables, thereby bolstering the reliability and accuracy of the findings [24].

Diagnostic accuracy of CECT in lymph node evaluation

Review of Studies

CECT has emerged as a promising tool for assessing lymph node involvement across various cancer types, encompassing colorectal, pancreatic, and oral cancers [28-30]. However, the diagnostic accuracy of CECT is contingent upon the particular cancer type and the location of the lymph nodes being evaluated. In the context of papillary thyroid carcinoma, a meta-analysis revealed that CECT yielded a sensitivity of 46% and a specificity of 88% in detecting cervical lymph node metastasis on a neck-level basis [28]. Notably, combining ultrasound with CECT enhanced the sensitivity to 51%, albeit with a slight decrease in specificity to 85% [28]. Similarly, in thyroid cancer, CECT demonstrated a sensitivity of 77%, specificity of 70%, and diagnostic accuracy of 74% on a per-level analysis [29]. For oral cancer, CECT exhibited a reliable detection of lymph node metastasis at IIa and IIb levels, with 88.46% and 92.86% of metastases being delineated, respectively [30]. However, in level III, only 62.96% of metastases were discerned on CECT, with a mere 44.44% exhibiting detectable enlargement [30]. In cervical cancer, a study reported a sensitivity of 31% for CECT in detecting nodal metastases on a patient-based level, accompanied by a negative predictive value of 80% and an area under the curve of 0.636 [31]. Notably, specificity and positive predictive value surged to 97% and 77%, respectively, when inconclusive lymph nodes were considered harmful [31]. While CECT demonstrates utility in evaluating lymph node involvement, its diagnostic accuracy fluctuates based on the specific cancer type and the location of the lymph nodes under scrutiny. The reported sensitivity ranges from 31% to 77%, while specificity spans from 70% to 97%, contingent upon the study design and analysis methodology [28-31]. Integrating CECT with complementary imaging modalities, such as ultrasound, may augment sensitivity but at the expense of reduced specificity [28].

Sensitivity and Specificity of CECT in Detecting Lymph Node Involvement

A study evaluating the detection of lymph node metastasis utilizing CECT and positron emission tomography-computed tomography (PET-CT) revealed that PET-CT exhibited a sensitivity of 78% and specificity of 92% for detecting lymph node metastasis, with an overall accuracy of 89% [32]. In another investigation comparing the sensitivity, specificity, and accuracy of arterial-phase CECT (A-CECT) and arterial and portal venous-phase CECT (A&C-CECT) for identifying metastatic lymph nodes, A-CECT demonstrated sensitivities ranging from approximately 73.2% to 75.3%. In comparison, A&C-CECT displayed a consistent sensitivity of 75.3%. The specificity of A-CECT varied between 92.2% and 97.2%, whereas A&C-CECT exhibited specificities ranging from 95.7% to 97.2% [33]. Furthermore, in a comparative analysis between CECT and CT perfusion for distinguishing benign from metastatic nodes, CECT showcased a sensitivity of 75%, specificity of 98.6%, and accuracy of 91.2% in discriminating between benign and metastatic nodes [34]. The sensitivity of CECT in detecting lymph node involvement spans approximately 73.2% to 75.3%, while specificity ranges from around 92.2% to 97.2%. These figures may vary slightly based on the specific study parameters and the type of cancer under evaluation.

Factors Influencing Diagnostic Accuracy

**Figure 1:**
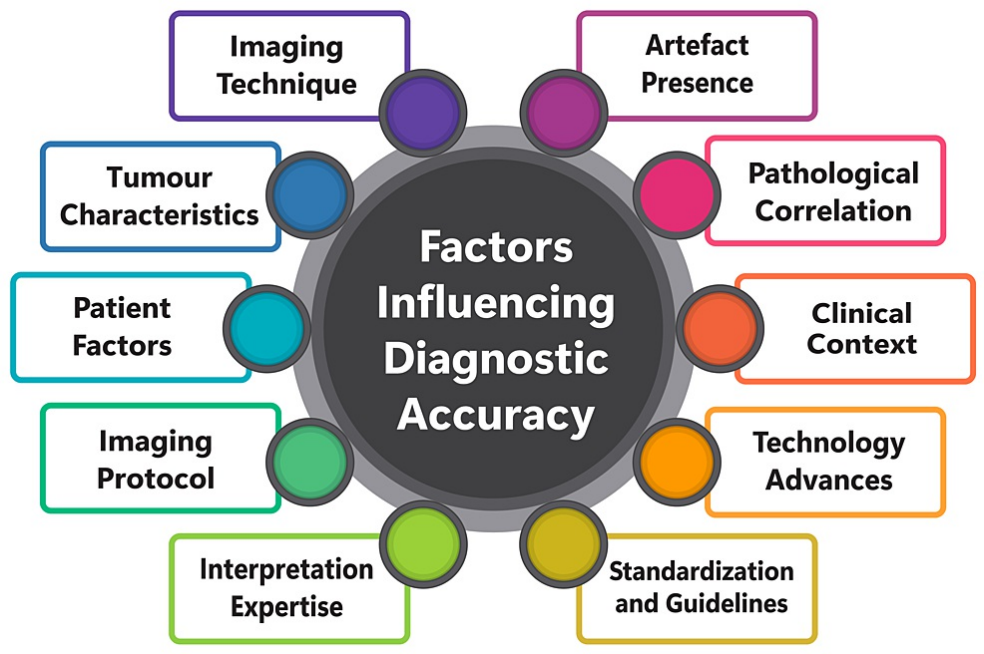
Factors influencing diagnostic accuracy Image Credit: Dr Akash Inamdar

The diagnostic accuracy of CECT in assessing various medical conditions is influenced by many factors, encompassing both technical specifications of CT systems and patient-specific characteristics. The research emphasizes the pivotal role of several key elements in shaping CECT's detectability performance. Technical factors, including CT system specifications, such as milliampere-second, peak kilovoltage, slice thickness, pitch, and beam quality, significantly impact the detectability performance of CT imaging systems [35]. Moreover, the advent of advanced technologies like multidetector CT (MDCT), dual-source CT (DSCT), and cone-beam CT (CBCT) has heightened the imperative to optimize image quality and assess potential reductions in low-contrast detail detectability [35]. Furthermore, clinicopathological characteristics are paramount in influencing the diagnostic accuracy of CECT, as evidenced by studies on cervical lymph node status in patients with oral squamous cell carcinoma [30]. Factors such as tumour localization, the type of neck dissection performed, and the appearance of lymph nodes on CT scans play a pivotal role in determining the reliability of CECT in detecting lymph node metastases [30]. In essence, the diagnostic accuracy of CECT hinges on a combination of technical factors related to CT system specifications and imaging parameters, as well as patient-specific characteristics such as tumour localization and neck dissection type. Consequently, comprehending and optimizing these factors are imperative for augmenting the diagnostic accuracy of CECT across various medical conditions [30]. Factors influencing diagnostic accuracy are shown in Figure 1.

Comparative analysis: CECT versus other imaging modalities

Magnetic Resonance Imaging (MRI)

CECT and magnetic resonance imaging (MRI) are prominent imaging modalities for assessing lymph node involvement across various cancers. Numerous studies have sought to compare the diagnostic accuracy of these techniques [36]. For instance, a study by Chen et al. focusing on nasopharyngeal carcinoma (NPC) revealed that MRI outperformed CECT in detecting retropharyngeal lymph node (RLN) metastases. Using a criterion based on a minimal axial diameter of ≥ 6 mm, MRI demonstrated a sensitivity of 0.71, specificity of 0.82, and a diagnostic odds ratio of 10.88 [37]. Similarly, Struckmeier et al. evaluated the diagnostic accuracy of CECT in assessing cervical lymph node status in oral squamous cell carcinoma. They categorized suspicious lymph nodes in CECT as accentuated (< 10 mm), enlarged (≥ 10 mm), and melted. The positive predictive value (PPV) of accentuated and enlarged lymph nodes in CECT was notably higher in poorly differentiated tumours than well- or moderately differentiated tumours [30]. In contrast, a meta-analysis by Miao et al. aimed to predict the metastatic status of lymph nodes in colorectal cancer using CECT. They achieved a sensitivity of 47.0% and a specificity of 80.9% for lymph nodes > 10 mm in size [38]. These studies collectively suggest that while CECT remains a valuable tool for evaluating lymph node involvement, MRI may offer superior accuracy in specific cancer types like NPC. Furthermore, the diagnostic performance of CECT can vary based on lymph node characteristics and tumour differentiation. Integrating CECT with advanced techniques such as radiomics and deep learning may further enhance its diagnostic capabilities [30,39]. Ultimately, the selection between CECT and MRI should be guided by the specific clinical context and the type of cancer being evaluated, considering the strengths and limitations of each modality.

Positron Emission Tomography (PET)

The comparison between CECT and positron emission tomography (PET) in imaging modalities for various medical conditions, including cancer, is a subject of considerable interest in medical research. Both imaging techniques present unique advantages and limitations in diagnosing and monitoring colorectal, pancreatic, and head and neck cancers [40,41]. Conversely, PET scans, as discussed in sources like Cancer.net, utilize a radioactive tracer to identify abnormal metabolic activity in the body, particularly in cancer cells [40]. PET scans are invaluable for assessing cancer stage, treatment response, and detecting distant metastases. In the realm of head and neck cancers, studies like the one highlighted in Medscape emphasize the significance of PET/CT scans in evaluating residual disease and lymph node involvement post-treatment [42]. PET/CT scans exhibit higher accuracy in detecting residual diseases in neck nodes compared to CECT, highlighting the complementary roles of these imaging modalities. While CECT offers detailed anatomical information, PET scans provide functional insights based on metabolic activity. The choice between CECT and PET imaging modalities often hinges on the specific clinical scenario, the type of cancer being evaluated, and the information necessary for an accurate diagnosis and treatment planning. Both modalities possess their strengths and limitations, and a comprehensive understanding of their capabilities is indispensable for optimal patient care in oncology and other medical fields [43].

Ultrasound (US)

The comparative analysis between CECT and other imaging modalities, specifically ultrasound (US), emphasizes distinct advantages and considerations for each technique. CECT, as elucidated in the provided sources, offers rapid imaging with immediate availability of images for interpretation, rendering it invaluable for assessing extra-hepatic tissues and organs [39]. It facilitates the characterization of focal liver lesions through enhancement patterns and enables volume estimation of the future liver remnant in cases of hepatic resection [39]. Nonetheless, CECT entails relatively high doses of ionizing radiation and necessitates the use of iodine-based contrast agents, which can trigger allergic reactions and anaphylactic shock, making it contraindicated for patients with prior allergic reactions or renal insufficiency [39]. On the flip side, contrast-enhanced ultrasound (CEUS) presents a non-invasive and cost-effective alternative to CECT and MRI for characterizing cystic renal lesions [44]. CEUS has demonstrated excellent sensitivity and negative predictive value, emerging as a promising screening tool for renal cystic lesions [44]. Moreover, CEUS proves advantageous in complex cystic renal mass surgeries, assisting in decision-making for curative surgeries and reducing the risk of unnecessary radical nephrectomy [45]. However, the efficacy of CEUS heavily relies on the skills of the performing physician, and there may be limitations concerning interrater reliability among readers and potential misdiagnosis of specific renal lesions [45]. While CECT offers immediate image availability and the ability to assess extra-hepatic tissues, it carries the drawbacks of ionizing radiation exposure and potential adverse effects from contrast agents. Conversely, CEUS presents a cost-effective and non-invasive option with remarkable sensitivity and negative predictive value, particularly in renal cystic lesions. The selection between CECT and CEUS hinges on the specific clinical scenario, necessitating a careful consideration of the advantages and limitations of each imaging modality [46].

Clinical implications and challenges

Impact on Treatment Planning

Studies have demonstrated that CECT has minimal dosimetric effects on dose calculations and monitor units in treatment planning for prostate, rectal, and head and neck cancers [47,48]. While contrast agents in CT imaging can influence dose distribution, the overall impact is considered clinically insignificant. CECT frequently alters clinical diagnoses with increased diagnostic certainty and prompts changes in initial treatment plans for many patients [49]. This emphasizes the importance of CECT in refining diagnoses and treatment strategies based on more accurate imaging information. Research has explored the value of contrast-enhanced PET/CT as a primary restaging tool in colorectal cancer, focusing on the impact of intravenous contrast on diagnostic confidence and patient management [50]. The findings suggest that utilizing contrast-enhanced PET/CT as the initial imaging modality can result in management changes and enhance diagnostic confidence in a substantial percentage of patients. Contrast-enhanced 4D-CT proves advantageous in accurately delineating tumours and assessing internal target volumes (ITV) [51]. This method enables precise target delineation and dose calculation, emphasizing reducing radiation exposure to patients and enhancing treatment accuracy. The impact of contrast-enhanced CT on treatment planning in various cancers, including colorectal cancer, is multifaceted. While it may have minimal effects on dose calculations in some cases, it plays a critical role in refining diagnoses, increasing diagnostic certainty, and guiding treatment strategies based on more accurate imaging information.

Limitations and Pitfalls of CECT

While CECT benefits lymph node assessment, its diagnostic accuracy may be constrained by size and morphology. For instance, in one study, lymph nodes more significant than 10 mm on CECT exhibited a sensitivity of only 47.0% and specificity of 80.9% for detecting metastases, emphasizing the importance of cautious interpretation and consideration of additional imaging modalities [52]. Concerns regarding radiation exposure arise with the use of CECT and other imaging modalities, especially in cases where multiple scans may be required for restaging and follow-up of colorectal cancer patients. Physicians must employ CECT solely when no alternative test or procedure can provide the necessary information and endeavour to minimize the radiation dose to the lowest level capable of delivering acceptable image quality [53]. Physicians sometimes fall into the trap of being misled by a negative CECT report, presuming that further definitive testing is unnecessary. However, CECT cannot substitute for examining spinal fluid or providing histologic evidence. Relying solely on CECT findings for precise diagnoses, such as "metastases" or "infarct," can lead to inaccuracies and potentially exacerbate tunnel vision rather than enhancing patient care [53]. The variability in guidelines for follow-up and restaging of colorectal cancer emphasize the imperative for standardized protocols and further research to optimize the utilization of CECT and other imaging tools [50]. Physicians should deploy CECT exclusively when no alternative test or procedure can furnish the requisite information and question the use of CECT when the indications appear inappropriate [53]. While CECT serves as a valuable tool for assessing lymph node involvement in colorectal cancer, its limitations and pitfalls, including suboptimal diagnostic accuracy, radiation exposure, misinterpretation of findings, and lack of standardization, emphasize the importance of judiciously considering its use and integrating it with other imaging modalities to ensure optimal patient care.

Future Directions and Emerging Technologies

Dual-energy CT (DECT) technology facilitates the simultaneous acquisition of images at two distinct energy levels, leading to enhanced tissue differentiation and improved detection of lymph node metastases [54]. Through leveraging the unique attenuation properties of tissues at different energy levels, DECT enhances the visualization of lymph nodes and holds the potential to enhance the diagnostic accuracy of CECT [54]. Integrating radiomics, which extracts quantitative features from CECT images, with machine learning algorithms offers promise in developing predictive models for lymph node metastasis [30]. By analysing many imaging features, radiomics models can uncover patterns that may not be visually discernible, thereby potentially refining the accuracy of CECT-based lymph node assessment [30]. Combining CECT with positron emission tomography (PET) in a single imaging modality, known as contrast-enhanced PET/CT (cePET/CT), has emerged as a promising approach in restaging colorectal cancer [50]. cePET/CT furnishes anatomical and functional information, potentially augmenting the detection of local recurrence and distant metastases compared to CECT alone [50]. Further research is warranted to delineate the optimal role of cePET/CT in managing colorectal cancer patients. Standardized protocols and guidelines are imperative to exploit the potential of CECT in lymph node assessment fully [50,52]. Consensus on factors such as contrast agent administration, imaging parameters, and interpretation criteria can mitigate variability and enhance the reproducibility of CECT findings across diverse institutions [50,52]. The advent of new contrast agents, particularly those founded on dual-energy CT technology, holds promise for augmenting the capabilities of CECT in lymph node evaluation [54]. These agents offer improved tissue differentiation and may enhance the detection of small or subtle lymph node metastases [54].

## Conclusions

In conclusion, this review highlights the pivotal role of CECT in evaluating lymph node involvement in colorectal cancer (CRC). CECT emerges as a valuable tool in the initial staging of CRC patients, providing high-resolution anatomical images and facilitating accurate identification of lymph node metastases and vascular invasion. However, while CECT demonstrates high sensitivity and specificity in detecting lymph node involvement, limitations such as suboptimal resolution for small lymph nodes and the potential for false-positive results underscore the need for cautious interpretation. Recommendations for clinical practice emphasize the integration of CECT into multidisciplinary treatment planning, alongside close collaboration between radiologists and clinicians to optimize imaging protocols and ensure an accurate interpretation of findings. Looking ahead, further research is warranted to refine imaging protocols, compare the effectiveness of CECT with emerging technologies, and validate its prognostic value through prospective studies correlating imaging findings with histopathological and long-term patient outcomes. By addressing these areas, CECT can continue to enhance the management of CRC by guiding treatment decisions and improving patient outcomes.
